# Transfer of Synthetic Human Chromosome into Human Induced Pluripotent Stem Cells for Biomedical Applications

**DOI:** 10.3390/cells7120261

**Published:** 2018-12-08

**Authors:** Sergey A. Sinenko, Elena V. Skvortsova, Mikhail A. Liskovykh, Sergey V. Ponomartsev, Andrey A. Kuzmin, Aleksandr A. Khudiakov, Anna B. Malashicheva, Natalia Alenina, Vladimir Larionov, Natalay Kouprina, Alexey N. Tomilin

**Affiliations:** 1 Institute of Cytology, Russian Academy of Sciences, 4 Tikhoretsky Ave., St-Petersburg 194064, Russia; s.sinenko@incras.ru (S.A.S.); e.skvortsova@incras.ru (E.V.S.); s.ponomartsev@incras.ru (S.V.P.); a.kuzmin@incras.ru (A.A.K.); 2 Division of Molecular and Radiation Biophysics, Petersburg Nuclear Physics Institute named by B.P. Konstantinov of National Research Centre “Kurchatov Institute”, Orlova Roscha 1, Gatchina 188300, Russia; 3 Developmental Therapeutics Branch, National Cancer Institute, Bethesda, MD 20892, USA; mikhail.liskovykh@nih.gov (M.A.L.); larionov@mail.nih.gov (V.L.); kouprinn@mail.nih.gov (N.K.); 4 Almazov National Medical Research Centre, 2 Akkuratova Str., St-Petersburg 197341, Russia; hudiakov.aa@gmail.com (A.A.K.); amalashicheva@gmail.com (A.B.M.); 5 Max-Delbruck Center for Molecular Medicine, 10 Robert-Rössle-Straße, 13125 Berlin, Germany; alenina@mdc-berlin.de; 6 Institute of Translational Biomedicine, St-Petersburg State University, 7-9, Universitetskaya nab., St-Petersburg 199034, Russia

**Keywords:** human artificial chromosome (HAC), alphoid^tetO^-HAC, induced pluripotent stem cells (iPSCs), microcell-mediated chromosome transfer (MMCT), cell reprogramming

## Abstract

Alphoid^tetO^-type human artificial chromosome (HAC) has been recently synthetized as a novel class of gene delivery vectors for induced pluripotent stem cell (iPSC)-based tissue replacement therapeutic approach. This HAC vector was designed to deliver copies of genes into patients with genetic diseases caused by the loss of a particular gene function. The alphoid^tetO^-HAC vector has been successfully transferred into murine embryonic stem cells (ESCs) and maintained stably as an independent chromosome during the proliferation and differentiation of these cells. Human ESCs and iPSCs have significant differences in culturing conditions and pluripotency state in comparison with the murine naïve-type ESCs and iPSCs. To date, transferring alphoid^tetO^-HAC vector into human iPSCs (hiPSCs) remains a challenging task. In this study, we performed the microcell-mediated chromosome transfer (MMCT) of alphoid^tetO^-HAC expressing the green fluorescent protein into newly generated hiPSCs. We used a recently modified MMCT method that employs an envelope protein of amphotropic murine leukemia virus as a targeting cell fusion agent. Our data provide evidence that a totally artificial vector, alphoid^tetO^-HAC, can be transferred and maintained in human iPSCs as an independent autonomous chromosome without affecting pluripotent properties of the cells. These data also open new perspectives for implementing alphoid^tetO^-HAC as a gene therapy tool in future biomedical applications.

## 1. Introduction

Gene therapy includes approaches to either correct gene function or provide a wild-type copy of a mutated gene. Traditional gene delivery and therapy techniques using viruses, plasmids, bacterial and yeast artificial chromosomes can cause random DNA insertions into the host genome, often leading to unpredicted transgene expression and cancer development in humans [1,2,3,4]. Included among the several disadvantages of commonly used virus-based delivery systems are low cloning capacity, unstable episomal maintenance, and the lack of long-term gene expression. Human artificial chromosomes (HACs) avoid these disadvantages and also provide the physiological expression of genes of interests as analogous to the native chromosome [5].

Originally and commonly used HACs have been built by a top-down approach by means of the truncation of various human chromosomes [6,7,8], referred to as “mini-chromosomes”. The presence of a functional kinetochore in HACs allows them to be maintained as additional functional chromosomes in mammalian cells over multiple cell divisions [9,10]. Such HACs were used as high capacity gene delivery vectors in mouse models of muscular dystrophies [11,12,13]. HACs carrying megabase-size DNA inserts were also employed for gene therapy in CYP-humanized and human antibody-producing mice [6,11,14,15,16]. 

Another type of HAC is synthesized based on the bottom-up approach. A novel synthetic HAC has recently been assembled from a synthetic α-satellite (alphoid) DNA array, in which the tetracycline operator (tetO) sequences were embedded allowing the binding of Tet repressor fusion proteins. This feature provides the opportunity to conditionally inhibit a kinetochore function, resulting in the loss of the HAC in dividing cells [17,18,19]. In addition to this feature, the alphoid^tetO^-HAC vector has several other advantages, such as a fully defined megabase-size synthetic alphoid DNA array lacking any cryptic transcripts [20,21]. The structural integrity of this HAC has been demonstrated during gene loading and its transfer into different host cells, along with the high mitotic and transcriptional stability of the transgenes over multiple rounds of cell division in culture [18,22]. Alphoid^tetO^-HAC shows several characteristics required for an ideal gene delivery vector and can be stably maintained in murine embryonic stem cells and their derivatives throughout mouse ontogeny [23]. In human cancer cell lines, like HeLa, the alphoid^tetO^-HAC has been reported to be rather unstable, however, tethering histone acetyl transferase (HAT) to the centromere can significantly stabilize the HACs [24]. The behavior of the alphoid^tetO^-HAC in pluripotent stem cells and human tissues remains uncharacterized.

Microcell-mediated chromosome transfer (MMCT) is the main technique to transfer HACs from donor to recipient cells [25,26]. Chinese hamster ovary (CHO) cells have traditionally been used as the most efficient chromosome donor cells because unlike most cell lines, they undergo repetitive hyperploidization in the presence of colcemid, leading to micronucleation and the formation of micronuclei. These are subsequently ripped off the donor cells, along with fragments of cytoplasm and cell membrane, by centrifugation in the presence of actin inhibitors (cytochalasin B or latrunculin B) acting as cytoskeleton disruptors [26,27]. The resulting cell fragments, referred to as microcells, are then fused with the target cells using different cell-fusion agents. Traditionally, polyethylenglicol has commonly been used as a cell fusion agent. However, several new commercially available transfection reagents and the modified cell fusion micronucleated technique have also been developed [23,28,29,30]. Due to low efficiency and an increased risk of cell aneuploidy induced by polyethylenglicol, a new modified MMCT method applicable to human cells that utilizes an envelope protein of murine leukemia retroviruses (MLVs), was introduced [31]. Amphotropic MLV infects mammalian cells via binding to the Pit-2 phosphate transporter, which is a highly conserved and ubiquitously expressed membrane protein in mammals. The modified MLV-envelop protein was successfully utilized as a one-directional fusion agent for donor CHO-derived microcells, showing an increased efficiency of the MMCT method (retro-MMCT) [31].

In this study, we took advantage of the retro-MMCT method to transfer an alphoid^tetO^-HAC expressing GFP into de novo derived human iPSCs (hiPSCs) for the first time. We analyzed mitotic stability of the alphoid^tetO^-HAC and showed that it can be maintained in these cells for multiple passages without affecting their pluripotent properties.

## 2. Materials and Methods

### 2.1. Lentivirus Preparations

Lentiviruses encoding pluripotency factors OCT4, SOX2, cMYC, and KLF4 (within polycistronic cassette) and rtTA were prepared. Namely, 293T cells were transfected with envelope-encoding pMD2.G (2.5 μg), packaging psPAX2 (7.5 μg), and either pHAGE2-tetO-miniCMV-hOct4-F2A-hKlf4-IRES-hSox2-E2A-hcMyc-W-loxP (OKSM), FUW-M2rtTA (hereafter rtTA), or EnvΔR-IRES-tdTomato plasmids (10 μg) by polyethylenimine hydrochloride (PEI 40 kDa, 40 μg) transfection method [32,33]. Lentiviruses in cell culture supernatant were collected and processed, as described elsewhere [33,34,35,36].

### 2.2. Reprogramming Human Mesenchymal Stem Cells with OKSM/rt-TA

Human mesenchymal stem cells (hMSC) were grown in a DMEM medium (Biolot, St-Petersburg, Russia) supplemented with 10% fetal bovine serum, FBS (HyClone, Thermo Fisher Scientific, Waltham, MA, USA), 100 U/mL penicillin, 100 mg/mL streptomycin, and 2 mM l-Glutamine (Gibco, Thermo Fisher Scientific, Waltham, MA, USA) [37,38]. The hMSCs were treated for mycoplasma by culturing them in media with 10 μg/mL ciprofloxacin (Myco-3, AppliChem, Darmstadt, EU) or 10 μg/mL Plasmocin (Invivogen, Toulouse, EU) for 1 passage (7 days). The cells were seeded, 6.5 × 10^4^ cells per well of 0.1% gelatin-pretreated 12 well-plate in the above media. Next day, the media was replaced with Opti-MEM media containing the packaged lentiviruses rtTA [39] and pHAGE2 [40], adjusting the multiplicity of infection (MOI) to 10–12 for each virus. Following 3–4 h of incubation, 500 μL Opti-MEM were added, and incubation was continued overnight. The next day, the media was changed to the above serum-containing media supplemented with 2 μg/mL Doxycycline (Dox). The media was changed every second day and after 6 days, the cultured cells were trypsinized, seeded onto 6-well plates pre-coated with L7 hPSC matrix, and cultured (37 °C, 5% CO_2_) in L7 hPSC BulletKit media (Lonza Group, Basel, Switzerland) containing 2 μg/mL Dox. The media was changed every third day. The hiPSC colonies were picked on day 28, expanded in the same medium, and frozen in liquid nitrogen.

The derivation of human mesenchymal stem cells (hMSC) was performed according to the Helsinki declaration, and approval was obtained from the local Ethics Committee of the Almazov National Medical Research Centre. Written informed consent was obtained from all subjects prior to tissue biopsy. The specific clinical research protocol was approved by the local Ethics Committee of the Almazov National Medical Research Center (Ethical permit number 12.26/2014).

### 2.3. MMCT into hiPSCs

MMCT was performed as previously described [15,23,27,31,41], with modifications. CHO cells carrying alphoid^tetO^-HAC-GFP [23] were transduced with the lentivirus-bearing EnvΔR-IRES-TdTomato transgene (MOI = 4, virus titer = 6 × 10^6^ tU/mL). The expanded CHO cells were cultured in T-25 flasks (Greiner) covered with 50 μg/mL collagen-I solution (Santa Cruz Biotechnology, Dallas, TX, USA) in a standard DMEM/F12 media (Biolot, St-Petersburg, Russia) supplemented with 10% FBS (HyClone, USA), 100 U/mL penicillin, 100 mg/mL streptomycin, 2 mM L-glutamine (Gibco, Thermo Fisher Scientific, Waltham, MA, USA), and 100 ng/mL colcemid (Wako Pure Chemical, Osaka, Japan), at 37 °C for 72 h with daily media change. The T-25 flask with micronucleated CHO cells was treated with DMEM containing 2 μM latrunculin B (Santa Cruz Biotechnology) and centrifuged at 8000× rpm at 34 °C for 1 h using an Avanti HP-26XP, JLA-10.500 rotor (Beckman Coulter Life Sciences, Indianapolis, IN, USA). The microcells fraction was collected as the total microcells pellets were consequently filtered through 8-, 5-, and 3-μm Whatman™ Nuclepore filters (Whatman, Piscataway, NJ, USA) and spun down by centrifugation at 3000 rpm for 5 min. The prepared microcell fractions were frozen with Cellbanker freezing media (Zenoaq, Tokyo, Japan) at −80 °C [41]. The hiPSCs were grown in mTeSR-1 media (Stemcell Technologies, Vancouver, BC, Canada) in 12-well plates up to 80–90% confluence. The cells were treated with Dispase (Stemcell Technologies, Vancouver, BC, Canada) for seven minutes and dissociated by fine micropipette tip scratching to produce small detached cell clusters. The hiPSCs were collected by centrifugation and softly suspended in 250 μL of pre-warmed mTeSR-1 media. Simultaneously, ¼ portion of the prepared microcells containing alphoid^tetO^-HAC-GFP were defrosted, washed in 10 mL of cold DMEM media, and suspended in 250 μL of pre-warmed mTeSR-1. Each 250 μL of hiPSC suspension and alphoid^tetO^-HAC-GFP microcell aliquots were mixed and carefully resuspended, spun down, and transferred into one well of 12-well plates covered with Matrigel (Corning, New York, NY, USA). The plates with the cell mixture were centrifuged (500 rpm, RT, 2 min), and incubated for 3–5 h at 37 °C in the standard CO_2_ incubator. The detached microcells were transferred into a separate Matrigel-covered well, and fresh mTeSR-1 media (1.5 mL) was added to the adhered hiPSCs. Next, the cells were grown for 24–48 h and passaged to 3 wells of 12-well plates covered with Matrigel. After separate colonies were grown, the GFP-positive ones were picked manually and transferred to separate wells of 12-well plates. The isolated GFP^+^ clones were grown to 25% confluency, then partly passed to fresh wells and partly frozen in 0.4 mL FreSR™-S media (Stemcell Technologies, Vancouver, BC, Canada). The hiPSCs were grown in various human embryonic stem cell media, including mTeSR-1, TeSR-E8 (Stemcell Technologies, Vancouver, BC, Canada), L7 hPSC BulletKit media (Lonza Group, Basel, Switzerland), StemFlex medium (Thermo Fisher Scientific, Waltham, MA, USA), cell matrices based on Matrigel (Corning, New York, NY, USA), and L7 hPSC Matrix (Lonza Group, Basel, Switzerland).

### 2.4. Preparation of Metaphase Spreads

The preparation of the metaphase nuclei was performed as previously described [23,27]. In brief, the exponentially growing HAC-carrying hiPSCs were incubated with 100 ng/mL colcemid (Wako Pure Chemical, Japan) for 4 h or overnight at 37 °C in 5% CO_2_ atmosphere. The cells were treated with hypotonic 0.56% KCl solution for 20 min and fixed by methanol/acetic acid solution (3:1, *v*/*v*). The prepared cell suspensions were placed dropwise on glass slides (Superfrost; Thermo Scientific, Darmstadt, Germany) and air-dried.

### 2.5. Fluorescence In Situ Hybridization with the PNA Probes

The slides with the metaphase spreads were treated with PBS for 15 min at RT, fixed in 4% paraformaldehyde (PFA), and washed four times for 7 min with PBS. The slides were consequently dehydrated with 70%, 90%, and 100% ethanol for five minutes each. A hybridization solution (20 μL) containing 10 M Tris–HCl pH 7.4, 70% formamide (Sigma-Aldrich, St. Louis, MO, USA), 5% dextran sulfate, 10 ng tetO PNA-FITC (Panagen Company, Bethel, PA, USA), and 10 ng telomere PNA-TRITS (Panagen Company, Bethel, PA, USA), was applied onto each slide and covered with cover glass. The slides were heated at 80 °C for 3 min and incubated for two to six hours at RT in darkness. The slides were then washed two times for 15 min with 70% formamide, 10 mM Tris–HCl (pH 7.4), 0.1% BSA, then washed three times for 5 min with 20 mM Tris–HCl (pH 7.4), 136 mM NaCl, 0.08% Tween-20, and finally rinsed in PBS. The slides were dehydrated, as indicated above, and mounted in Vectashield media containing 4′,6-diamidino-2-phenylindole (DAPI) (Santa Cruz Biotechnology, Dallas, TX, USA). Images were captured using the EVOS Cell Imaging Systems (Thermo Fisher Scientific, Waltham, MA, USA).

### 2.6. FACS Sorting of hiPSCs

The hiPSCs were grown in six-well plates in standard StemFlex medium/Matrigel conditions until 90% confluency, treated with L13 hPSC passaging solution (Lonza Group, Basel, Switzerland), washed with DMEM/F12, and resuspended in 1 mL StemFlex medium. Cell sorting of the GFP-positive and GFP-negative hiPSCs was done using flow cytofluorimeter EPIX XL (Beckman Coulter, Brea, CA, USA). The sorted cells were collected in PBS, centrifuged, and seeded on single wells of 12-well plates pre-covered with Matrigel.

### 2.7. Karyotype Analysis

The logarithmically grown hiPSCs (confluence 50–60%) were treated with 400 ng/mL colcemide for 16 h and trypsinized. The metaphase spreads were prepared as described above. The karyotype of the hiPSCs was defined using G-banding metaphase chromosomes analysis at a resolution of 400 bands, with twenty metaphase plates being analyzed.

### 2.8. Cell Immunostaining 

Immunostaining of the cells was done as described previously [33]. The cells grown attached to the culture surface were fixed in 4% PFA in PBS, washed with PBS, and treated for 30 min with a blocking PBS solution of 1% BSA, 2% nonimmune sheep serum, and 0.1% Tween-20. The cells were next incubated with mouse antibodies to OCT4 (Santa Cruz Biotech, Dallas, TX, USA), SOX2 (NBC, Astana, Kazakhstan), NANOG (NBC, Astana, Kazakhstan), and KLF4 (Santa Cruz Biotech, Dallas, TX, USA), washed several times in 0.1% Tween20-PBS, and incubated with secondary antibodies conjugated with Cy-3 (Jackson ImmunoResearch, West Grove, PA, USA). Subsequently, the cells were washed in 0.1% Tween20-PBS, counterstained with DAPI, and embedded under coverslips into an anti-fading media.

### 2.9. Southern-Blot Hybridization Analysis

Southern-blot hybridization was performed with a ^32^P-labelled DNA probe [27]. The genomic DNA from 5 × 10^5^ cells was digested by *Spe*I in an agarose plug. The digested CHEF DNA (CHEF Mapper, Bio-Rad Laboratories, Hercules, CA, USA) was gel-separated (5–250 kb range, 16 h run), transferred onto membrane (Amersham Hybond-N^+^), and hybridized with a 201-bp YAC/BAC DNA probe specific for alphoid^tetO^-HAC. The DNA probe was PCR-amplified from the genomic DNA in the presence of ^32^P-labeled dNTPs, using the 5′-GGGCAATTTGTCACAGGG-3′ and 5′-ATCCACTTATCCACGGGGAT-3′ primers. The blot was pre-hybridized for two hours at 65 °C in Church’s buffer containing 7% SDS and 0.5 M Na-phosphate buffer supplemented with 100 µg/mL salmon sperm DNA, then hybridized overnight at 65 °C with heat denatured 201-bp YAC/BAC DNA probe. The blot was washed twice in 0.05% SDS, 2× SSC for 10 min at RT, then twice in 0.05% SDS, 2× SSC for five minutes at 60 °C, twice in 0.05% SDS, 0.5× SSC for 5 min at 60 °C and twice in 0.05% SDS, 0.25× SSC for 5 min at 60 °C, developed for 24–72 h at −80°C.

### 2.10. Treatment of Cells with Inhibitors of Chromatin Modifiers

The Alphoid^tetO^-HAC-GFP hiPSCs were cultured for 24 h in mTeSR-1 media in the presence of DNA methyltransferase inhibitor 5-Aza-2’-deoxycytidine (AZA) (Sigma-Aldrich, St. Louis, MO, USA) or histone deacetylase inhibitor trichostatin A (TSA) (Sigma-Aldrich, St. Louis, MO, USA) at concentrations of 5–10 μM and 0.5 μM, respectively. The Alphoid^tetO^-HAC-GFP hiPSCs were cultured for 72 h in the presence of 100–400 nM AZA or 19–38 nM TSA. The GFP-expression in the living cells was monitored by fluorescent and phase contrast light microscopy (EVOS FL Auto Imaging System, Thermo Fisher Scientific, Waltham, MA, USA).

The Alphoid^tetO^-HAC-GFP hiPSCs were cultured for 24 h in mTeSR-1 media in the presence of DNA methyltransferase inhibitor 5-Aza-2’-deoxycytidine (AZA) (Sigma-Aldrich, St. Louis, MO, USA) or histone deacetylase inhibitor trichostatin A (TSA) (Sigma-Aldrich, St. Louis, MO, USA) at concentrations of 5–10 μM and 0.5 μM, respectively. The Alphoid^tetO^-HAC-GFP hiPSCs were cultured for 72 h in the presence of 100–400 nM AZA or 19–38 nM TSA. The GFP-expression in the living cells was monitored by fluorescent and phase contrast light microscopy (EVOS FL Auto Imaging System, Thermo Fisher Scientific, Waltham, MA, USA).

## 3. Results and Discussion

### 3.1. Reprogramming Human Endometrial MSCs in Lonza’s cGMP Culture Conditions

**Figure 1:**
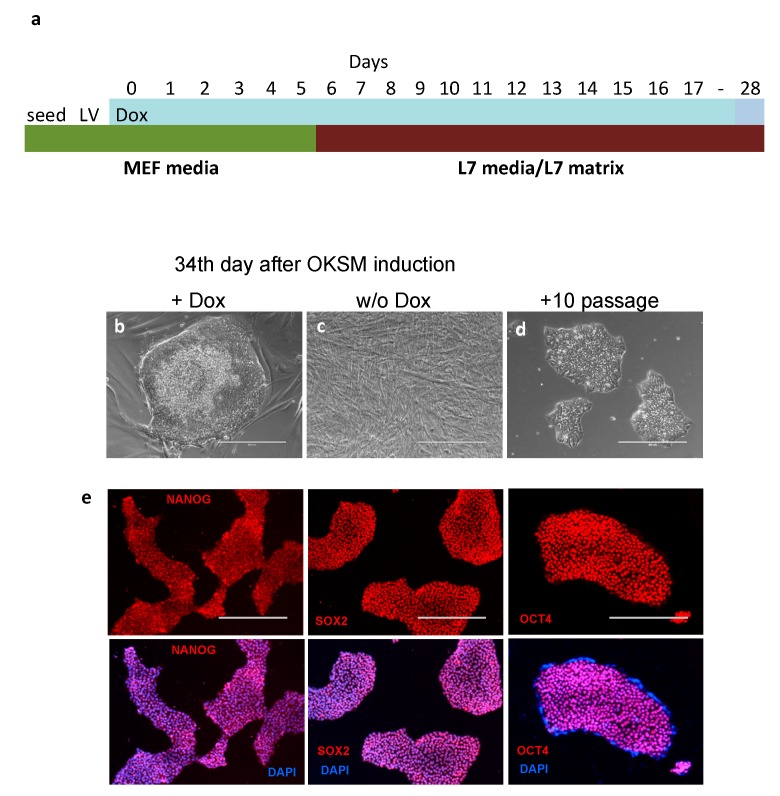
Generation of human pluripotent stem cells (hiPSCs) from human mesenchymal stem cells (hMSCs). (**a**) OKSM cassette expression was induced by Dox (2 μg/mL) on day 0, and on day 6 cells medium was changed to the L7. Distinctive hiPSC clones were observed on day 28. Representative images of (**b**) hiPSC clone on day 34 after OKSM induction, (**c**) uninfected hMSCs cultured for the same period of time, and (**d**) hiPSCs after 10 passages in culture. (**e**) hiPSCs cultured for 7 passages retain pluripotent characteristics, such as expression of NANOG, SOX2, and OCT4 marker genes (red). Scale bar represents 400 μm.

To generate hiPSCs in conditions compliant with current Good Medical Practice (cGMP), we used the culture reagents from Lonza [42,43]. These reagents, originally developed for the generation of hiPSCs from human peripheral blood mononuclear cells, were applied in this paper for the reprogramming of human mesenchymal stem cells (hMSCs). The hMSCs were taken from healthy women and grown in a standard serum-containing media (see Materials and Methods). The cells were simultaneously infected with lentiviruses containing tetO-driven Oct4, Klf4, Sox2, cMyc (OKSM) polycistronic cassette, and reverse tetracycline-controlled transactivator (rtTA) constructs [40]. Following the addition of Doxycycline (Dox), the cells were trypsinized, transferred onto fresh wells pre-covered with L7 matrix, and cultured in Lonza L7 hPSC medium (Figure 1a). On day 28 of the reprogramming, four iPSC clones were picked and expanded (Figure 1b). Although we found that the Matrigel/mTeSR-1 medium was superior for hiPSCs maintenance, L7 matrix/L7 medium conditions were preferred because they were compliant with cGMP standards. Importantly, the use of the OKSM polycistronic construct is very suitable for hiPSCs generation from hMSCs. Antibody staining confirmed that newly derived hiPSCs express the pluripotency markers NANOG, SOX2, and OCT4 (Figure 1e). Thus, the feeder cell- and serum-free L7 matrix/L7 media is both compliant with cGMP and very suitable for hMSCs reprogramming to pluripotent state. Notably, a case of successful reprogramming of hMSCs to iPSCs using a cell-feeder based conventional method has been previously reported [44]. At the same time, the reprogramming of human dermal fibroblast, using the same Lonza’s culture conditions and OKSM construct, has not been successful, as the fibroblasts showed a dramatic overgrowth. This culture media system was originally designed to reprogram human peripheral blood mononuclear cells under hypoxic conditions. Because we used the normoxic conditions and hMSCs at rather advanced passages, this probably yielded a relatively low number of hiPSC clones. We believe that the method could be significantly improved by applying hypoxic conditions (3–5% O_2_) and by using hMSCs at early passages. Additionally, we have found that the obtained hiPSCs can be maintained for multiple passages in TeSR-E8 and StemFlex human embryonic stem cell media with the use of both Matrigel and L7 matrixes. In summary, we applied in this study, for the first time, feeder-/serum-free cGMP-compliant conditions to reprogram hMSCs into hiPSCs.

### 3.2. MLV Envelope Protein-Mediated Transfer of Alphoid^tetO^-HAC to Human iPSCs.

**Figure 2:**
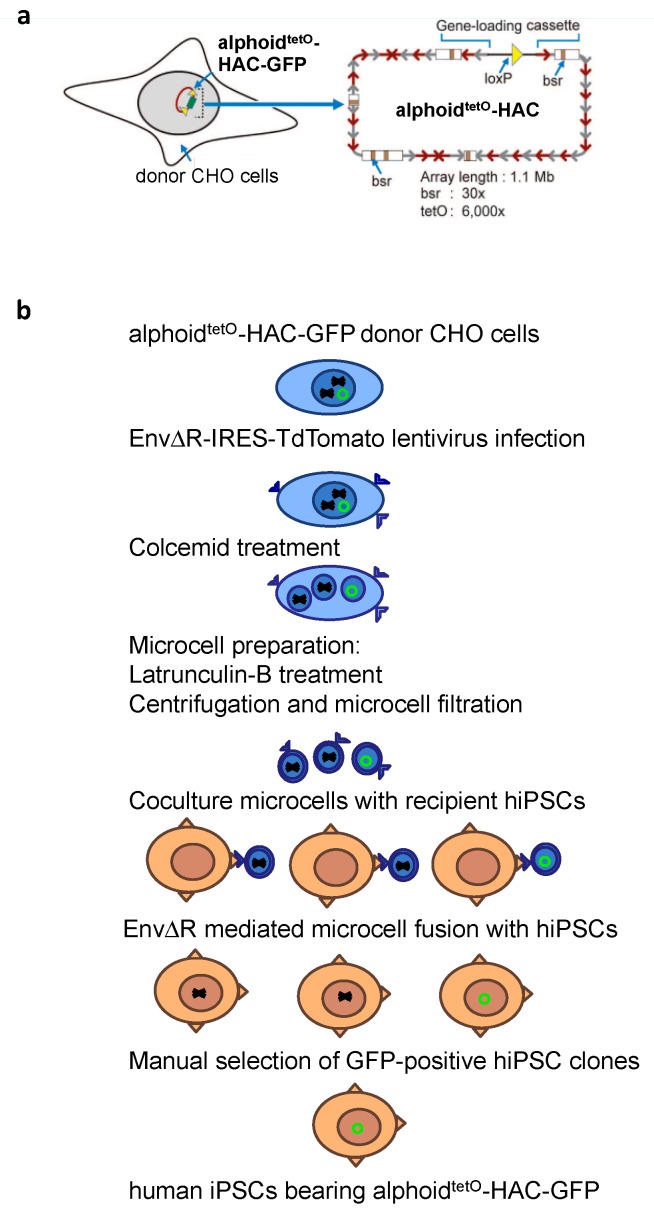
Scheme of microcell mediated chromosome transfer (MMCT) method used in this paper. (**a**) Schematic representation of alphoid^tetO^-HAC-GFP vector. (**b**) MMCT of the alphoid^tetO^-HAC-GFP into human pluripotent stem cells (iPSCs) with the use of mouse leukemia virus envelop protein (EnvΔR) as a cell fusion agent and latrunculin B as a cytoskeleton disruptor.

**Figure 3:**
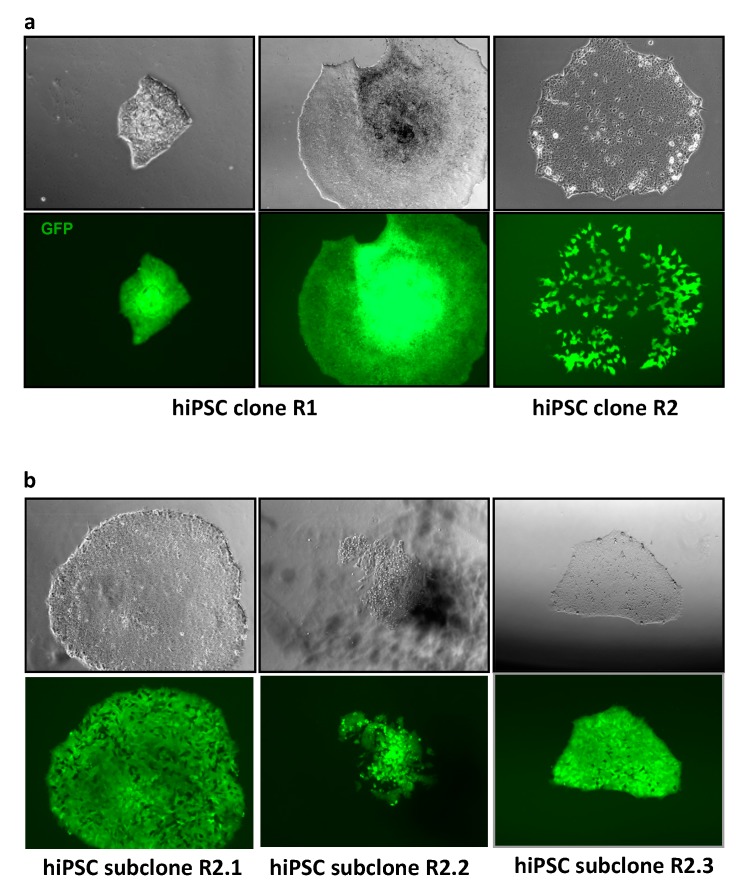
Evaluation of GFP expression stability in primary alphoid^tetO^-HAC-GFP human pluripotent stem cells (hiPSC) colonies. (**a**) Following the retro-MMCT procedure, GFP-positive colonies became visible on day 5–7; they were picked up and expanded as R1 and R2 alphoid^tetO^-HAC-GFP hiPSC clones. (**b**) The mixed clone R2 was further sub-cloned, giving rise to the homogeneous GFP-positive subclones R2.1, R2.2, and R2.3.

**Figure 4:**
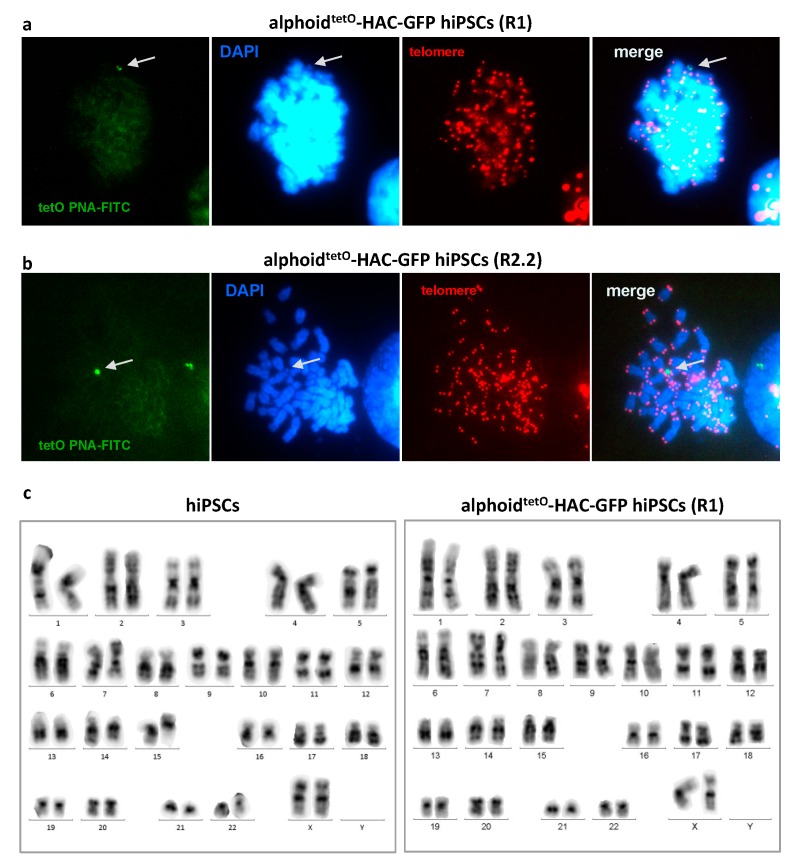
Alphoid^tetO^-HAC-GFP is maintained as an independently replicating chromosome in human pluripotent stem cells (hiPSCs), as revealed by fluorescence in situ hybridization (FISH) analysis using the tetO-PNA-FITC as a probe. The human artificial chromosome (HAC) (arrow) within the alphoid^tetO^-HAC-GFP carrying iPSC clone R1 (**a**) and sub-clone R2.2 (**b**) colocalizes with diamidino-2-phenylindole (DAPI) (blue) but not with the telomere labeling PNA-TRITS probe (red) specific exclusively for host chromosomes. (**c**) Representative results of karyotype analysis of the initial hiPSCs and the alphoid^tetO^-HAC-GFP hiPSCs (clone R1), showing normal 46XX karyotype.

Conventional polyethylenglycol- and hemagglutinating virus of Japan (HVJ) envelope-based MMCT methods were highly inefficient for the transfer of the HACs into embryonic stem cells and iPSCs of the human origin. The efficiency of these methods was significantly improved by several recent modifications [27,31,45]. In this study, we applied the novel method developed by Suzuki et al [31] to transfer GFP-expressing alphoid^tetO^-HAC to hiPSCs (Figure 2). In addition, latrunculin B was used as a more efficient actin inhibitor for cytoskeleton disruption during microcells preparation [27]. Hamster donor CHO cells bearing alphoid^tetO^-HAC-GFP were infected with the EnvΔR protein-expressing lentivirus, resulting in >90% of the cells being positive for the EnvΔR protein expression. The prepared microcells were frozen and then fused with the hiPSCs. Two hiPSC clones bearing alphoid^tetO^-HAC-GFP were selected based on GFP expression, and subsequently expanded (referred to as R1 and R2 clones, Figure 3a). One of these clones (R2) was found to be a mixture of GFP-positive and GFP-negative cells. Therefore, it was further sub-cloned, resulting in three independent subclones homogeneously expressing GFP (R2.1, R2.2, and R2.3) (Figure 3b). Based on fluorescence in situ hybridization (FISH) assay, the alphoid^tetO^-HAC-GFP was maintained as an independent chromosome in the analyzed hiPSC clones (Figure 4). To analyze whether or not the HAC has undergone structural rearrangements during the course of the MMCT from hamster CHO to hiPSCs, Southern blot hybridization was carried out with genomic DNA possessing the alphoid^tetO^-HAC-GFP (clone R1), digested by SpeI endonuclease. This nuclease cuts the RCA/SAT43 vector sequence once but does not have a recognition site in the 1.1 Mb alphoid DNA array of alphoid^tetO^-HAC [20]. The original alphoid^tetO^-HAC carries 47 copies of the RCA/SAT43 vector used for the assembly and propagation of the synthetic alphoid DNA array [19]. SpeI-digested genomic DNA was separated by CHEF and hybridized with the probe specific to the tetO-alphoid sequence (see Materials and Methods for details). As seen in Figure S1, multiple identical bands of different sizes were observed on the Southern blot after SpeI digestion of CHO and hiPS cells. Thus, no detectable changes in the HAC structure were detected by Southern blot following the MMCT transfer of the HAC from CHO to hiPSCs.

Unlike mouse ESCs and iPSCs, their human counterparts are maintained in primed pluripotency state culture conditions which require more complex culture media and an extra-cellular matrix to sustain multiple cell passages. We have found that, contrary to all cell lines tested so far, hiPSCs are not susceptible to blasticidin (Bsd) selection. More specifically, hiPSCs bearing alphoid^tetO^-HAC-GFP, which contains multiple copies of the Bsd resistance gene [20], died within 48 h in the presence of 5 μg/mL Bsd. At the same time, both the parental iPSCs and the alphoid^tetO^-HAC containing hiPSCs could survive for at least 120 h in media containing 3.5 μg/mL Bsd. Thus, the sorting approach based on the living marker (GFP) is the method of choice for the selection of alphoid^tetO^-HAC in hiPSCs cells.

### 3.3. Alphoid^tetO^-HAC Maintenance in Human Induced-Pluripotent Stem Cells

**Figure 5:**
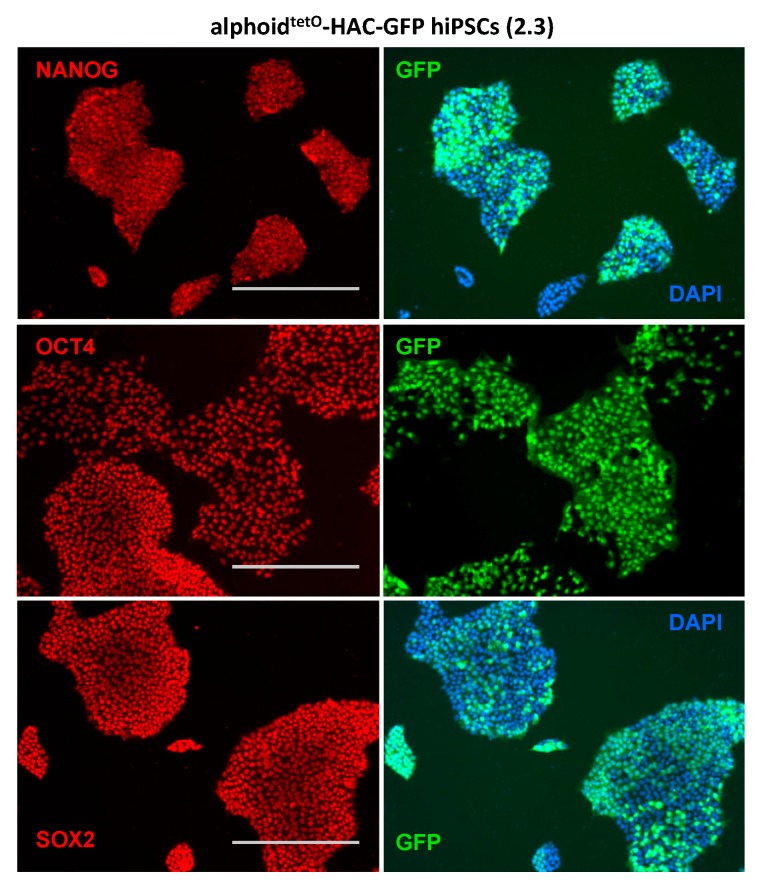
Human pluripotent stem cells (hiPSC) bearing alphoid^tetO^-HAC-GFP cells express pluripotency markers. Alphoid^tetO^-HAC-GFP hiPSCs maintained for over 5 passages remain pluripotent as they express OCT4, NANOG, and SOX2 markers (red), indicated on the panels. Scale bar, 400 μm.

The hiPSCs carrying alphoid^tetO^-HAC-GFP remained similar to the parental wild-type hiPSCs with regard to growth and pluripotent stem cell characteristics for at least 15 passages. Importantly, even after such a prolonged time in culture, the hiPSCs showed stable karyotype, colony morphology, and expression of stem cell marker genes SOX2, OCT4, and NANOG (Figure 5 and Figure S2). G-banding analysis of the alphoid^tetO^-HAC-GFP bearing hiPSC clones revealed a normal 46XX karyotype with no apparent chromosomal abnormalities (Figure 4C). Interestingly, we noticed that after the first passage of alphoid^tetO^-HAC-GFP hiPSCs both GFP-positive and GFP-negative cells could be found (see Figure S3). After several passages, however, the number of GFP-negative cells of initially homogenous GFP-positive population of alphoid^tetO^-HAC bearing hiPSCs is significantly increased (up to 50% cells become GFP negative). This might be the result of either HAC loss or the silencing of GFP expression in some cells, or both. To address this, we separated the GFP-positive and GFP-negative cells by FACS and followed GFP expression in these sub-populations. Importantly, both sub-populations behaved similarly in standard culture conditions. However, even after one passage, GFP-negative cells emerged in population enriched for GFP-positive cells (Figure S3). On the other side, GFP-positive cells also emerged in GFP-negative population (Figure S3), suggesting that the former cells contain the HAC with silenced GFP whose expression is re-initiated after 1–2 passages. To further clarify the reason for the loss of GFP expression, we performed FISH analysis of the GFP-sorted cells, using the HAC-specific labeled probes (see Materials and Methods). More than 85% of the cells (from 25 metaphase spreads) exhibited a positive FISH signal in the population of GFP-positive cells (Figure S4). These results indicate that silencing of the GFP-expressing cassette within the HAC may occur. It was shown that epigenetic chromatin modifiers, such as inhibitors of DNA methyltransferase (5-Aza-2’-deoxycytidine or AZA) and histone deacetylase (Trichostatin A or TSA), can reactivate GFP-expression from alphoid^tetO^-HAC [46]. Treatment of GFP-positive cells by AZA or TSA resulted in a significant increase of GFP expression. However, neither of these inhibitors promoted an increase of GFP expression in the hiPSCs population sorted for GFP-negative cells (Figure S5). These data confirm that the alphoid^tetO^-HAC-GFP was lost in these cells and that HAC is mitotically unstable in hiPSCs.

## 4. Conclusions

De novo assembled alphoid^tetO^-HAC represents a promising new generation of high-capacity episomal vectors for biomedical applications [17,20,47]. In this study, we took advantage of the recent progress made in the MMCT methodology and succeeded in delivering the alphoid^tetO^-HAC into the hiPSCs which are currently considered to be the most clinically promising cell types in regenerative medicine. However, there are several limitations of the HAC-based technology that need to be resolved before it can be implemented in hiPSC-based clinical practice. These limitations include the low efficiency of HAC formation, the complex repeated DNA structure of the HACs, significant challenges in the amplification of a large amount of the HAC vector outside of eukaryotic cells, poor efficiency of the HAC delivery into target tissues or organs [6,7,48,49,50] and, as shown in this paper, insufficient mitotic stability of the HAC in hiPSCs. However, understanding the mechanism of HAC propagation in hiPSCs is critical for its application in gene therapy. Addressing the latter problem is a highly relevant pursuit in our future research.

## Supplementary Materials

The following are available online at http://www.mdpi.com/2073-4409/7/12/261/s1. Figure S1: Southern blot analysis of integrity of the alphoid^tetO^-HAC-GFP in hamster CHO cells (clone 38-18) and after MMCT into hiPSCs (clone R1). Figure S2: hiPSCs bearing the alphoid^tetO^-HAC-GFP express pluripotency markers. Figure S3: Silencing of GFP expression in a fraction of alphoid^tetO^-HAC-GFP hiPSCs.**** Figure S4: FISH analysis of FACSed alphoid^tetO^-HAC-GFP hiPSCs revealed loss of the HAC in the population of GFP-negative cells. Figure S5: AZA and TSA treatment of alphoid^tetO^-HAC-GFP hiPSCs does not increase number of GFP-positive cells.
