# Supplementary material for: Transfer of Synthetic Human Chromosome into Human Induced Pluripotent Stem Cells for Biomedical Applications
Source: Cells. 2018 Dec 8;7(12):261. doi: 10.3390/cells7120261 (PMC6316689; doi:10.3390/cells7120261)
Supplement: Supplementary file 1 [file cells-07-00261-s001.pdf]

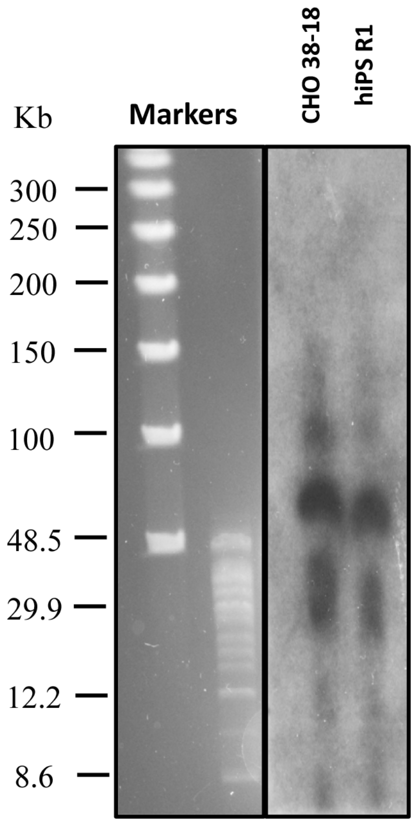

Figure S1. Southern blot analysis of integrity of the alphoid^{tetO}-HAC-GFP in hamster CHO cells (clone 38-18) and after MMCT into hiPSCs (clone R1). Genomic DNA possessing the HAC isolated from CHO and hiPSCs was digested with SpeI endonuclease which cuts the HAC only in the vector part, and separated by CHEF gel electrophoresis (range 10−300 kb). The transferred membrane was hybridized with the tetO-alphoid probe (see Materials and Methods). M - Markers: CHEF DNA Size Lambda Ladder (BIO-RAD) and 8-48 kb DNA size standards (BIO-RAD)

alphoid^{tetO}-HAC-GFP hiPSCs (R1)

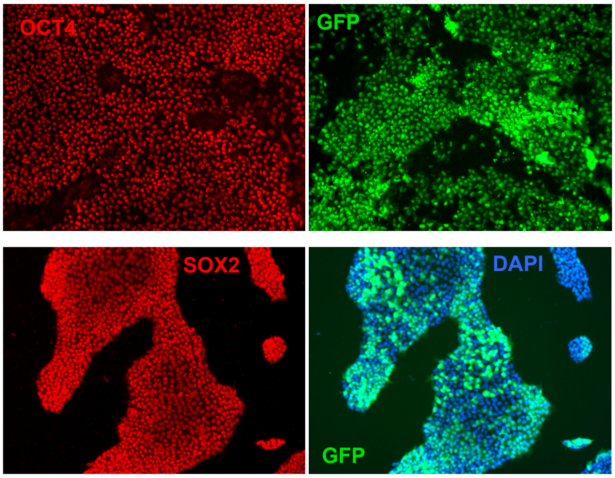

Figure S2. hiPSCs bearing the alphoid^{tetO}-HAC-GFP express pluripotency markers. alphoid^{tetO}-HAC-GFP hiPSCs (clone R1) maintained for over 5 passages in ES cell culture conditions remain pluripotent as they express OCT4, and SOX2 markers (red), indicated on the panels. Scale bar, 400 mm.

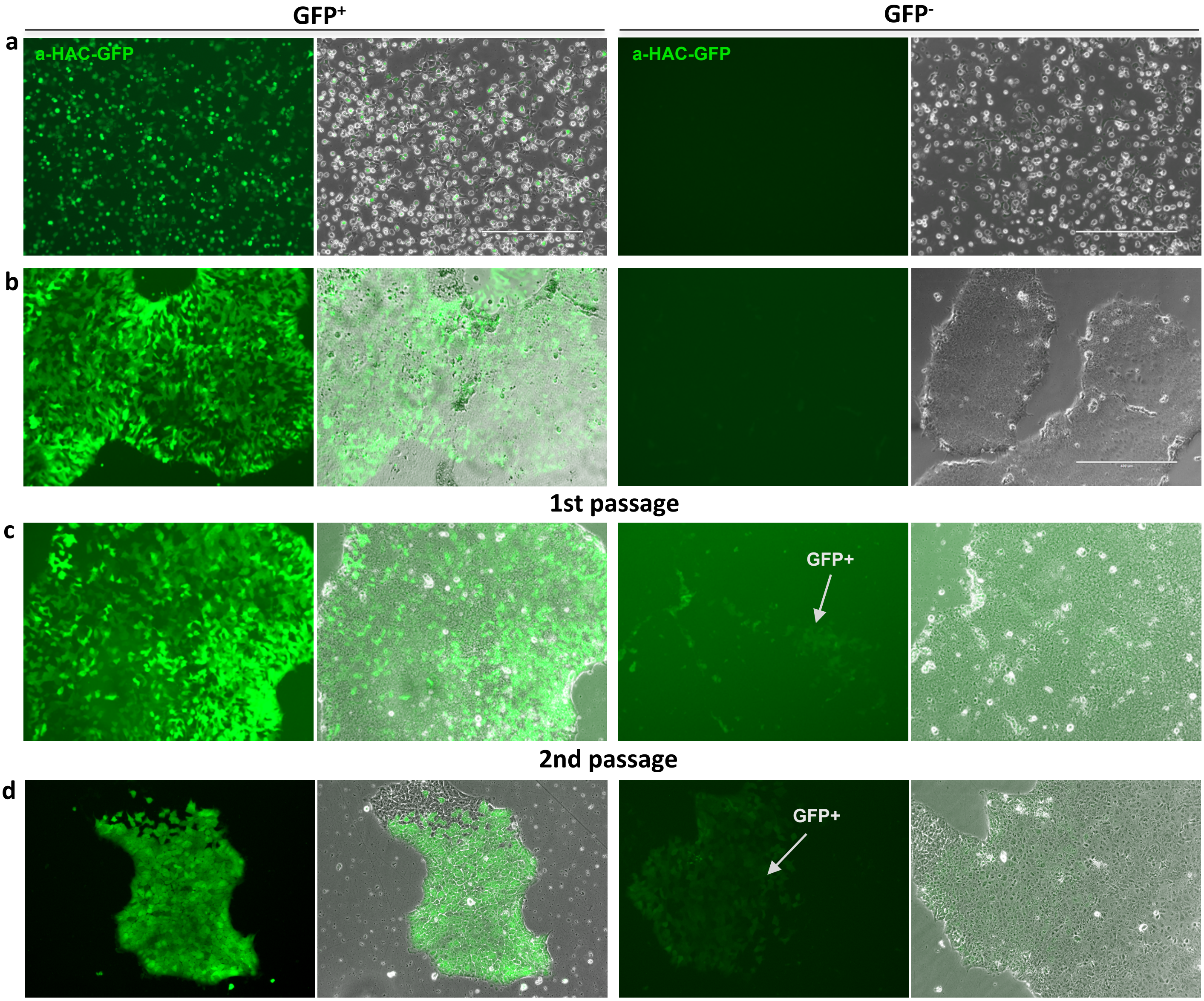

Figure S3. Silencing of GFP expression in a fraction of alphoid^{tetO}-HAC-GFP hiPSCs. (a) FACS of alphoid^{tetO}-HAC-GFP hiPSCs (R2.3) after 5 passages, images of GFP^{+} and GFP$^{–}$ cell fractions seeded on Matrigel-coated wells after cell sorting. (b) Images of the above GFP^{+} and GFP$^{–}$ on day 7 after seeding, (c, d) rare cells show onset of GFP expression in GFP$^{–}$ cell fractions already after 1-2 passages (arrow)

	Containing HAC	Lacking HAC	HAC bearing cells(%)
GFP+ hiPSCs	12	2	85%
GFP- hiPSCs	2	8	20%

- Examples of metaphase spreads see below

hiPSCs with alphoid^{tetO}-HAC-GFP (R2.3), GFP^{+} population

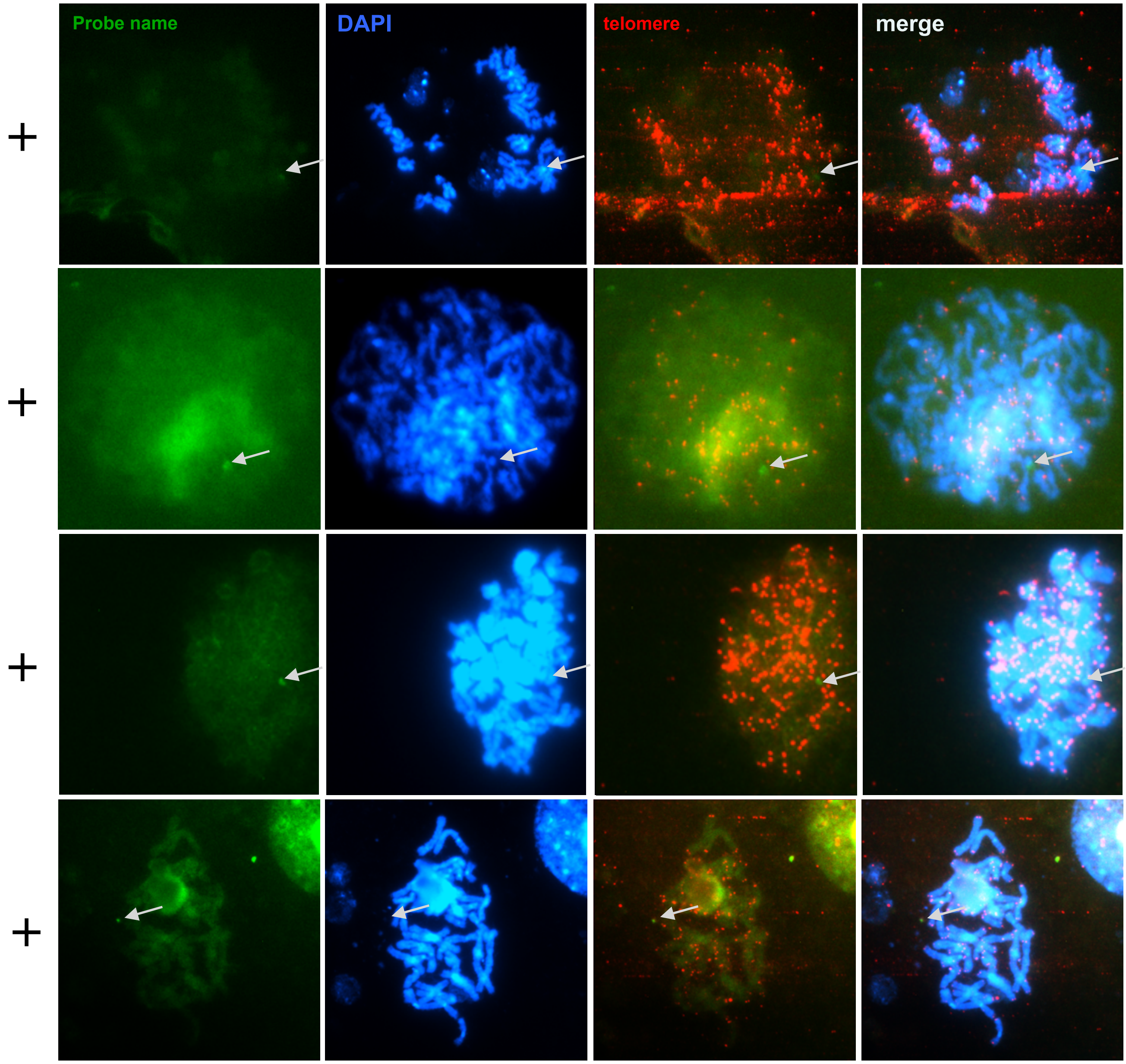

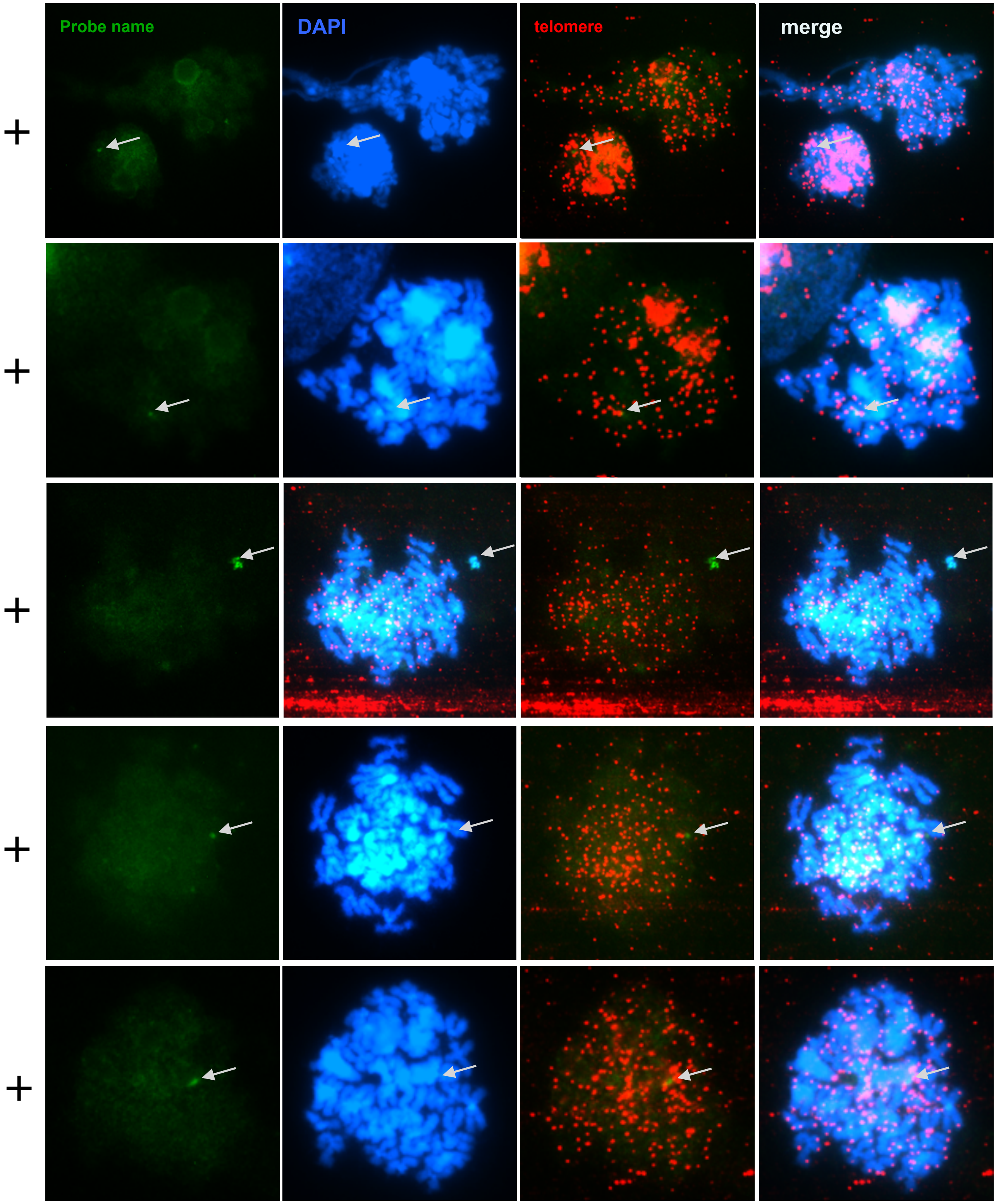
Probe name
DAPI
telomere
merge
+
+
+
+
+

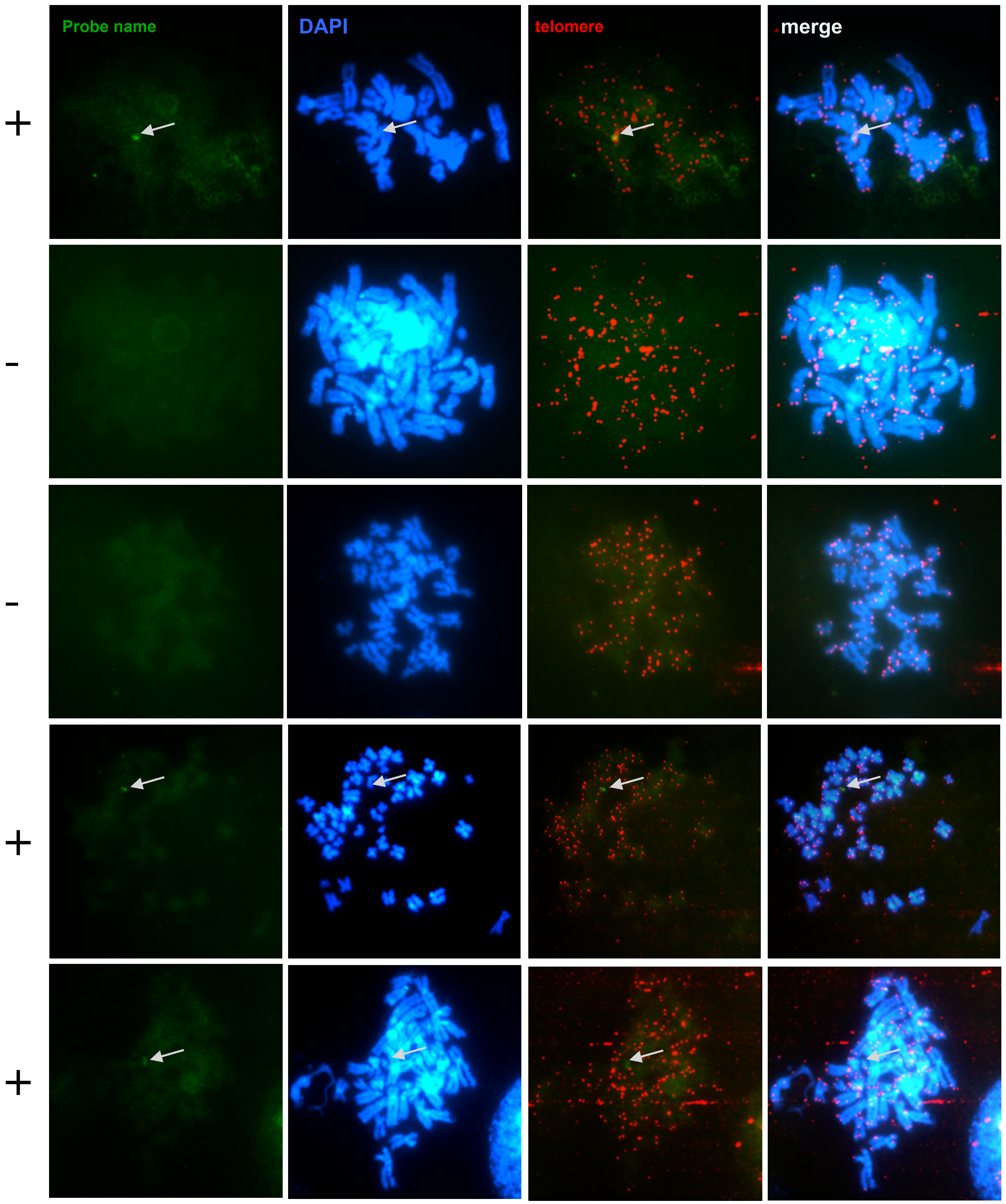
Probe name
DAPI
telomere
merge
+
-
-
+
+

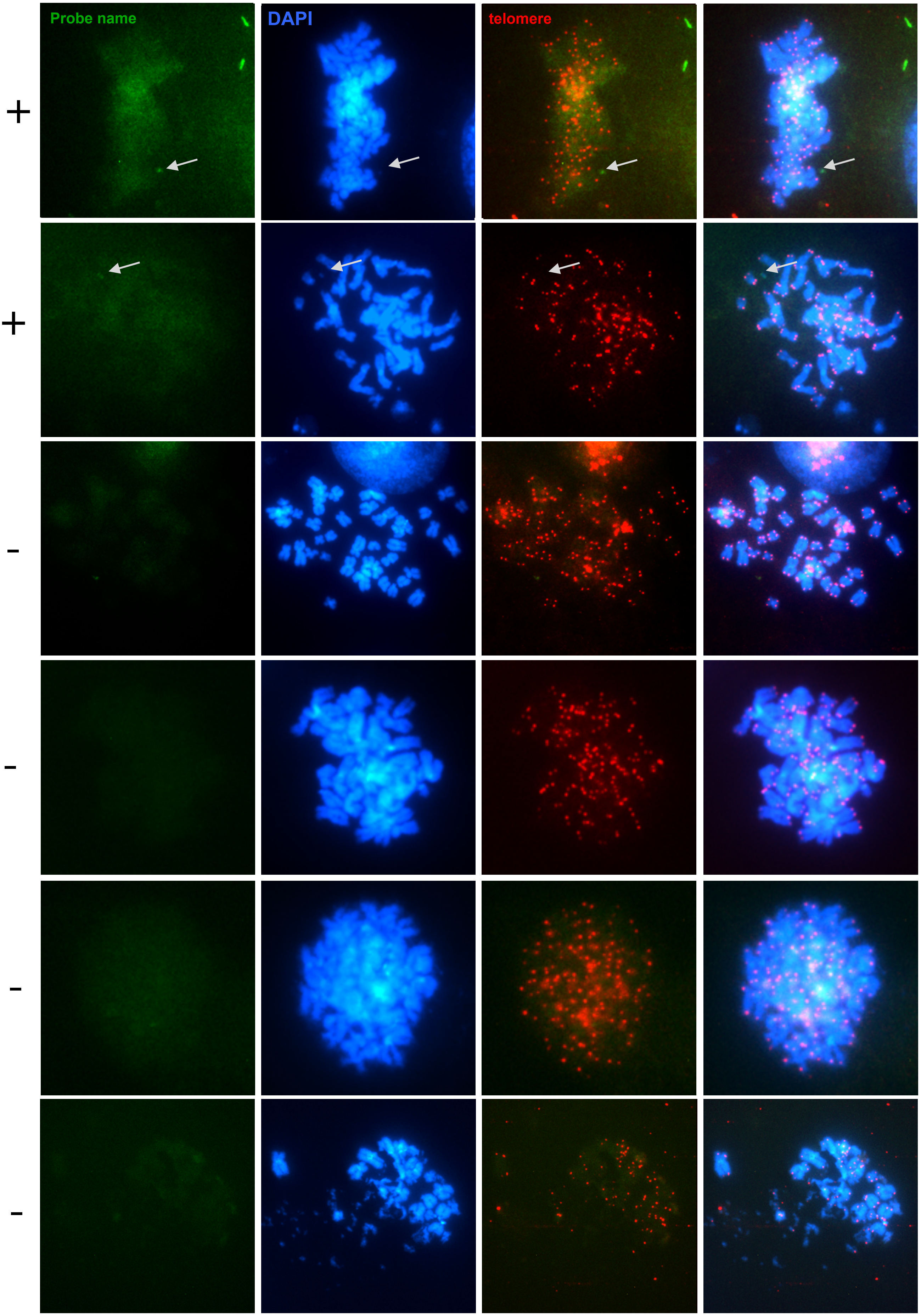
Probe name
DAPI
telomere
+
+
-
-
-
-

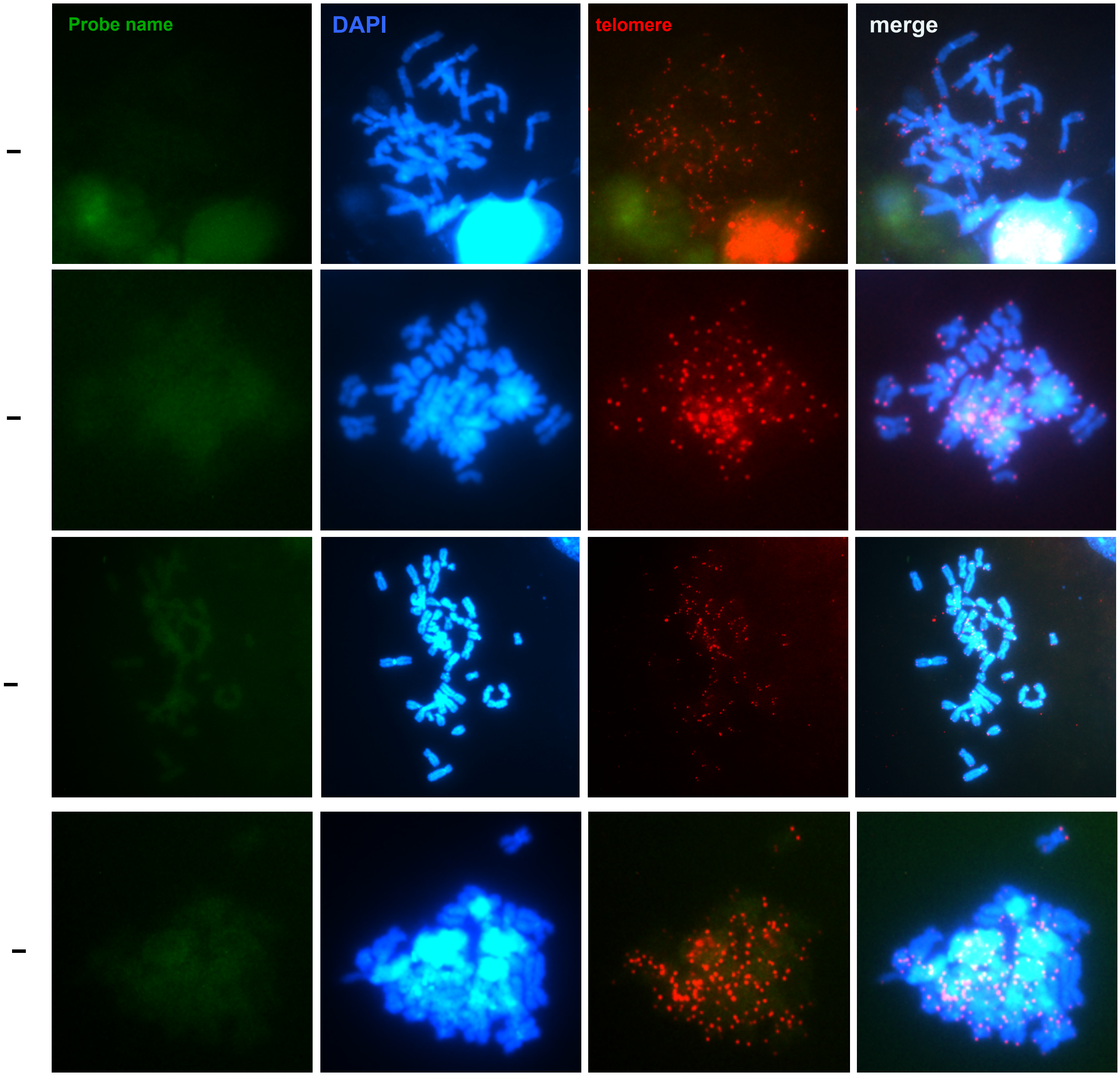

Figure S4. FISH analysis of FACSed alphoid^{tetO}-HAC-GFP hiPSCs revealed loss of the HAC in the population of GFP-negative cells. GFP-positive and GFP-negative cells were separated by FACS, and cultured for 7 days before the FISH assay. Summary of metaphase spread counts represented on the Table S1. Examples of metaphase nuclei from populations of GFP-positive and GFP-negative cells shown at passage 5 following the FACS. Alphoid^{TetO} HAC-GFP is revealed with tetO PNA-FITC as a probe. The HACs (green, arrows) colocalizes with DAPI (blue) but not with PNA-TRITS labeled telomere probe (red), which is specific exclusively for host chromosomes.

a

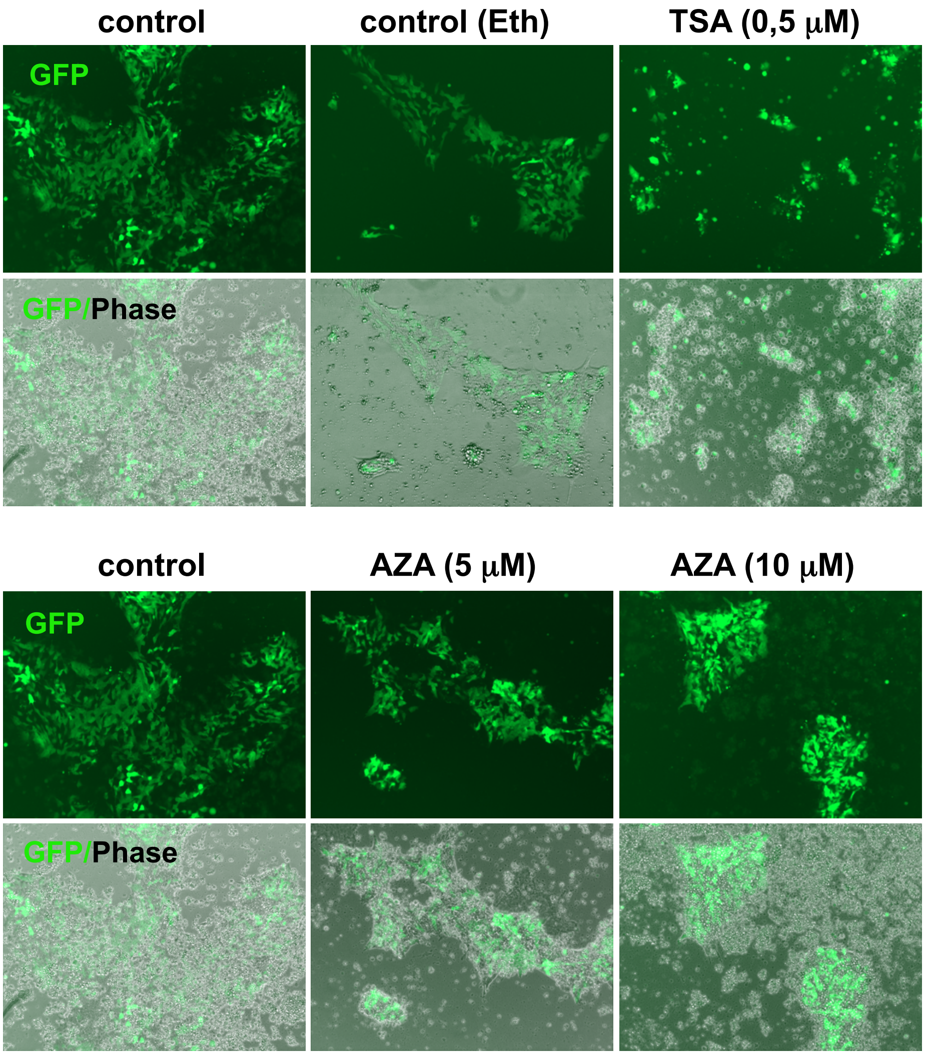
control
control (Eth)
TSA (0,5 μM)
GFP
GFP/Phase
control
AZA (5 μM)
AZA (10 μM)
GFP
GFP/Phase

b

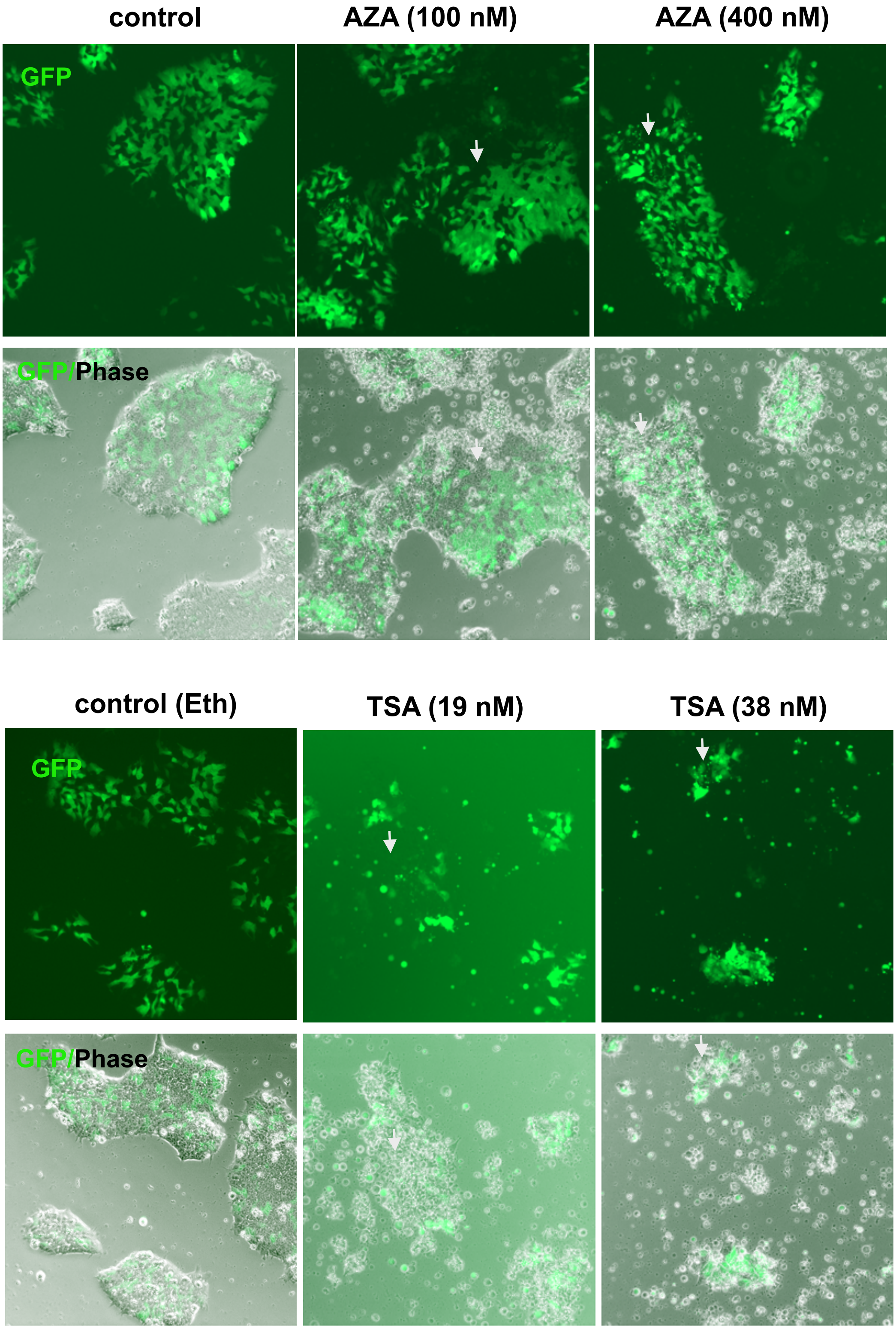
control
AZA (100 nM)
AZA (400 nM)
GFP
GFP/Phase
control (Eth)
TSA (19 nM)
TSA (38 nM)
GFP
GFP/Phase

c

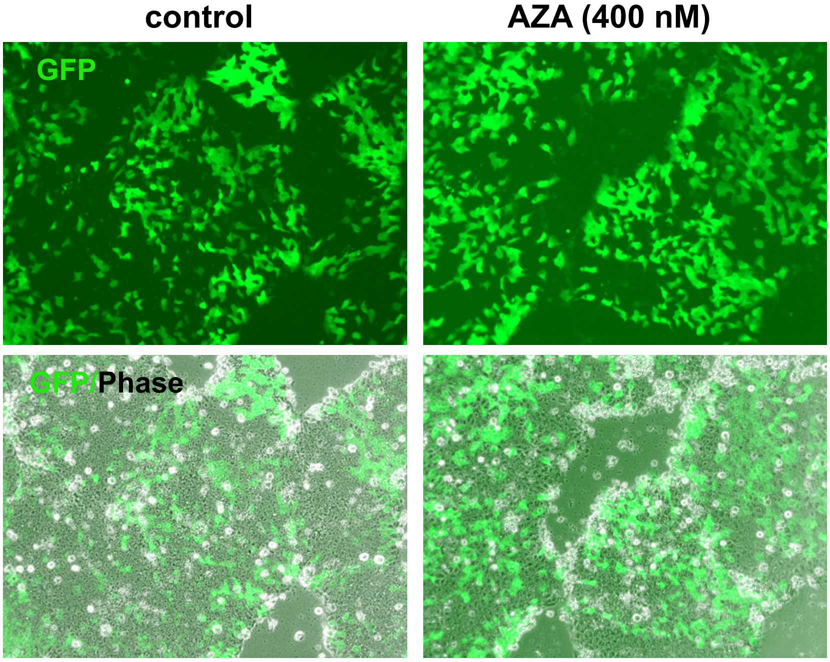

Figure S5. AZA and TSA treatment of alphoid^{tetO}-HAC-GFP hiPSCs does not increase number of GFP-positive cells. (a) 24-hour treatment of the cells with indicated high concentrations of AZA (5, 10 uM) and TSA (0,5 uM) does not increase number of GFP positive cells, however, it further increases GFP expression in already GFP-positive cells. (b) 24-hour cultivation of alphoid^{tetO}-HAC-GFP hiPSC with low concentrations of AZA (100, 400 nM) and TSA (19, 38 nM) does not affect number of GFP-positive cells. GFP-negative cells indicated by arrows. Control untreated cells and treated with ethanol vehicle (Eth) are indicated. (c) Prolonged cultivation (72 hrs) of alphoid^{tetO}-HAC-GFP hiPSCs with 400 nM AZA does not affect number of GFP-positive cells if compare with control.
